# Long-Term Phellodendri Cortex Supplementation in the Tiger Grouper (*Epinephelus fuscoguttatus*): Dual Effects on Intestinal Health Revealed by Transcriptome Analysis

**DOI:** 10.3390/life13122336

**Published:** 2023-12-13

**Authors:** Yan Cai, Huizhong Shi, Yu Zheng, Yongcan Zhou, Weiliang Guo, Jingqiu Liao, Shifeng Wang

**Affiliations:** 1 Collaborative Innovation Center of Marine Science and Technology, Hainan University, Haikou 570228, China; azsure_caiyan@hainanu.edu.cn (Y.C.); 15666933219@163.com (H.S.);; 2 Hainan Provincial Key Laboratory for Tropical Hydrobiology and Biotechnology, School of Marine Biology and Fisheries, Hainan University, Haikou 570228, China; 3 Guangxi Academy of Sciences, Nanning 530007, China

**Keywords:** transcriptome, Phellodendri Cortex, *Epinephelus fuscoguttatus*, long-term supplementation

## Abstract

The tiger grouper (*Epinephelus fuscoguttatus*), an important mariculture fish in Southeast Asia, faces increasing health issues in recent years. Phellodendri Cortex (PC) is a traditional Chinese herbal medicine that exhibits a variety of beneficial effects on tiger groupers. The effects of PC, however, varies with the period of dietary intervention. This study aims to investigate the long-term effects of 1% PC supplementation on tiger groupers, focusing on growth, immunity, disease resistance, and intestinal gene expression. The tiger groupers (with an initial mean weight of 27.5 ± 0.5 g) were fed with a diet of Phellodendri Cortex supplementation and a control diet for 8 weeks. Our results indicate that the long-term PC supplementation did not affect growth or *Vibrio* disease resistance in tiger groupers. However, the transcriptome analysis revealed potential damage to the structural and functional integrity of the groupers’ intestines. On the other hand, anti-inflammatory and cathepsin inhibition effects were also observed, offering potential benefits to fish enteritis prevention and therapy. Therefore, long-term PC supplementation in grouper culture should be applied with caution.

## 1. Introduction

The tiger grouper (*Epinephelus fuscoguttatus*) is widely farmed in the subtropical sea areas of the Indian Ocean and the Pacific Ocean [1]. However, the increase in the prevalence of diseases associated with bacteria, viruses, and parasites has hampered the development of grouper culture, resulting in mass economic losses in the industry [2,3,4,5]. The current therapeutic agents for the above-mentioned diseases are mainly antibiotics and chemical agents. However, the resulting adverse effects, such as drug resistance and environmental pollution, have made these disease control approaches undesirable [6]. Medicinal plants and their byproducts, which are natural and environmentally friendly, have shown great potential to become alternatives to antibiotics and chemicals, since many of them have been demonstrated to be effective for disease control and prevention [7,8].

Phellodendri Cortex (PC, “Huangbo” in Chinese) is the dried bark of *Phellodendron amurense* Ruprecht. As one of the most commonly used traditional Chinese herbal medicines, PC has been used for the treatment of diarrhea, hemorrhoids, toxicity, swollen red eyes, mouth sores, and other symptoms in China for thousands of years [9]. Berberine is one of its main components and its active ingredient [10], and recent studies have demonstrated that PC with its berberine has anti-bacterial, anti-inflammatory, and antioxidant effects. It can also have hypoglycemic, anti-tumor, and neuroprotective effects. Because numerous studies have shown that only extremely low amounts of berberine (about 0.36%) can enter the systemic circulation [11], we suspected that the majority of the regulatory effect of PC functioned through the intestine.

The intestine is a multifunctional organ in fish. As the largest and most-complex secretory organ in the body of a fish, it is critical for digestion, absorption, and the regulation of the metabolism [12]. The intestinal tract is also an important immune organ, since it provides biological, physical, and chemical barriers that enable fish to fight against bacterial invasion or absorption of toxic substances [13,14]. Intestinal health and normal functionality are critical for fish health. In recent years, to reduce costs, the use of considerable amounts of plant-derived proteins, oil, starch, and gluten in aquafeeds has become the norm in carnivorous fish farming [15,16,17,18]. However, carnivorous fish, such as Atlantic salmon and groupers, are known to develop intestinal damage and inflammation when they consume high levels of terrestrial plant components (such as soybean oils and protein). Fish that are farmed in aquaculture are susceptible to many environmental stresses induced by the poor water quality and high stocking densities that are common in many farming practices. For example, an increase in ammonia nitrogen, nitrites, and nitrate contents can cause irreversible damage to intestinal function [19,20]. The intestine, being a member of the gut–brain axis, exhibits abnormal molecular responses and its functions are jeopardized when exposed to environmental stresses [21,22,23]. With the intensity in development of intensive aquaculture over the last decade, intestinal damage and inflammation, which is a non-infectious disease that leads to serious economic loss, has become a major issue linked to carnivorous fish farming [24].

Fortunately, intestinal health is usually manageable to some degree. Studies have shown that microbial preparations (probiotics, prebiotics, or symbionts) can alleviate intestinal damage and inflammation through manipulation of the intestinal micro-ecosystem [25]. In addition, a number of additives and drugs can also regulate the expression of tight junction-related, metabolism-related, or immune-related genes in the intestine.

A previous study in our laboratory revealed that a one-week supplement of 1% PC (in relation to the basal diet) can significant downregulate the gene expressions of IL-8 and IgM in the head kidney of tiger groupers [26]. PC might, therefore, be a promising short-term feed additive to reduce inflammatory signs in tiger groupers. However, the long-term effect of the PC supplement in grouper fish and how it might affect the gene expression of the intestine remain unknown.

In this study combining enzyme activity analysis and transcriptome analysis, we explored the long-term (8-weeks) effects of the PC supplement on the antioxidant capacity, non-specific immunity, intestinal gene expression, and disease resistance of tiger groupers. This present research will further our understanding of the long-term effects of PC in tiger groupers and will provide insights into the molecular regulatory mechanism of PC on intestinal genes in teleost fish.

## 2. Materials and Methods

All the experimental procedures of this study were approved by the Hainan University Animal Use and Care Committee (No. HNUAUCC-2020-00011).

### 2.1. Preparation of PC Extract and Experimental Diets

Phellodendri Cortex (PC) was bought from a local, traditional Chinese drugstore in Hainan, China. After being washed with tap water and dried in an oven at 60 °C, the herb was ground into powder and sieved through a 40-mesh sieve (with an aperture of 0.425 mm). Then, 100 g of PC powder was suspended in 1000 mL of deionized water at room temperature for 72 h. Afterwards, the powder suspension was centrifuged at 9000× *g* for 5 min, and the supernatant was collected. The resulting supernatant (a water extract of PC) was stored at 4 °C for subsequent use.

Commercial feed (comprising 51.16% of crude protein, 13.45% of crude lipid, and 7.41% of ash; from Tongwei^®^, Tongwei Co., Ltd., Chengdu, China) was used as the basal diet. To prepare the experimental diet, the PC water extract was incorporated into the basal diet through spraying 10 mL of the PC water extract (corresponding to 1 g of PC powder) on every 100 g of the basal diet. The resulting experimental diet had a crude drug–basal diet ratio of 1% (*w/w*). The experimental diets were packed and stored at 4 °C until use. New batches of the experimental diets were prepared each week to ensure the freshness of the experimental diets.

### 2.2. Rearing

In total, 240 juvenile tiger groupers, *Epinephelus fuscoguttatus*, were obtained from a local hatchery at Haikou City, Hainan province. These juveniles (with a mean body weight of 27.5 ± 0.5 g) were acclimated to the experimental conditions for two weeks before the feeding experiment began. During the acclimation time, fish were fed the control diets. At the end of the acclimation period, the tiger groupers were separated into two groups with 40 fish per tank in three replicates. The control group and the experimental group were fed with the control diet and the experimental diet, respectively. Fish were fed twice daily (at 10:00 and 16:00), at a feeding rate of 2% of body weight/day for 56 days (8 weeks). All tanks were maintained under natural lighting. During the entire feeding trial period, the flowing water rate was kept at 0.2 L/min and the water quality was maintained within the following ranges: a temperature of 22 ± 3 °C, a pH of 7.3 ± 0.3, a dissolved oxygen concentration of 5.8 mg/L, and a salinity of 30.

### 2.3. Sampling

At the end of the 8-week feeding trial, blood and intestine samples were taken from five fish that were randomly chosen from each tank. Before sampling, the fish were fasted for 24 h. The fish were first anesthetized with MS222 (tricaine methanesulfonate, 100 mg/L), and then their body weight and body length were measured. Afterwards, blood was collected from the caudal artery and allowed to set in a 4 °C refrigerator for 24 h. The blood was centrifuged at 4000 rpm for 10 min, and then the supernatant was collected and stored at −80 °C for the determination of non-specific immunity parameters. After the blood sampling, the fish liver, spleen, and mid-intestine were all excised. The liver and spleen were weighed to measure the hepatosomatic index and the spleen-somatic index. The mid-intestine was frozen in liquid nitrogen for 24 h, then transferred to −80 °C until the RNA extraction.

### 2.4. Growth Performance, Hepatosomatic Index, and Spleen-Somatic Index

At the beginning and end of the 8-week feeding trial, the total weight of all the fish in each tank was measured, and the average fish weight (the total weight/the total number of fish) of each tank was used as one replicate for the average fish weight of each group. Then the livers and spleens of these fish were excised and weighed. The weight gain rate (WGR, %), specific growth rate (SGR, %), hepatopancreas somatic index (HSI, %) and spleen-somatic index (SSI, %) were calculated as follows:

WGR (%) = [100 × (final weight − initial weight)/initial weight];

SGR (% day^−1^) = 100 × [ln (final weight) − ln (initial weight)]/days;

HSI (%) = (liver weight/body weight) × 100;

SSI (%) = (spleen weight/body weight) × 100.

### 2.5. Biochemical Analysis

The amounts of superoxide dismutase (SOD, Cat. No. A001–3), lysozyme (LZM, Cat. No. A050-1), catalase (CAT, Cat. No. A007-1), alkaline phosphatase (ALP, Cat. No. A059-2), and total antioxidant capacity (T-AOC, Cat. No. A015-2) of the serum samples were measured using microplate methods, following the instructions of the commercial assay kits (Nanjing Jiancheng Bioengineering Institute, Nanjing, China). These indices were measured to reflect the changes in the non-specific immunity indices.

### 2.6. Challenge Test

A fish pathogen *Vibrio harveyi* strain GDH11385 was isolated from a previous study [27] and maintained as frozen stock in a −80 °C freezer. For the challenge test, the frozen stock was resuscitated. The bacterial suspension was adjusted to a concentration of 8.46 × 10^6^ CFU/mL.

On the day of the challenge test (Day 56), 30 fish from each group were intraperitoneally injected with 80 μL of the *Vibrio harveyi* strain GDH11385 at a dose of 8.46 × 10^6^ CFU/mL. At the same time, another 30 fish from each group were injected with 80 μL of PBS to serve as the control group. The applied LD_50_ value of the *V. harveyi* strain GDH11385 (8.46 × 10^6^ CFU/mL × 80 μL ÷ 38 g fish body weight = 1.7 × 10^4^ CFU/g fish body weight) was based on the previous work of our laboratory [27], and a preliminary LD50 test was conducted before the formal challenge experiment. The mortality was observed and recorded for 7 days after the challenge tests. All dead and surviving fish in the challenge tests were examined for bacteria to verify the presence of the pathogen.

### 2.7. RNA Extraction, cDNA Library Construction, and Transcriptome Sequencing

RNA extraction, cDNA library construction, and transcriptome sequencing were all conducted by Meiji Biotechnology Co., Ltd. (Shanghai, China). Total RNA was extracted from the middle intestines of the fish by using an RNA extraction kit (Promega, Shanghai, China) according to the manufacturer’s instructions. The purity and the concentration of the RNA were examined with a NanoDrop 2000 spectrophotometer (NanoDrop Technologies, Wilmington, DE, USA). The quality of the RNA was assessed with 1% agarose gel electrophoresis. The RIN score was measured with an Agilent 2100 bioanalyzer (Agilent, Shanghai, China). It was only when the quantity of the obtained RNA was more than 1 g and of high quality (OD260/280 ≥ 1.8, OD260/230 ≥ 1.0) that it could be used to construct a single cDNA library. The eukaryotic mRNA was enriched using magnetic beads with Oligo (dT). The obtained mRNA was randomly broken-up by adding a fragmentation buffer, and then the mRNA fragments of about 300 bp were separated using magnetic beads. Afterwards, these mRNA fragments were used as the template for the first-strand cDNA synthesis using random hexamer primers and reverse transcriptase, followed with second-strand cDNA synthesis. The double-stranded cDNA was repaired by adding an End Repair Mix and adding an A tail. Finally, ten cDNA libraries (five libraries for each group) were constructed and sequenced on the Illumina Novaseq 6000 platform at Majorbio Bio-Pharm Technoloay Co., Ltd. (Shanghai, China), following the manufacturer’s instructions.

### 2.8. De Novo Assembly and Gene Functional Annotation

The raw sequencing reads were filtered by applying fastx_toolkit_0.0.14 (http://hannonlab.cshl.edu/fastx_toolkit/, accessed on 30 May 2019) and fastq_0.19.5 (https://github.com/OpenGene/fastp, accessed on 30 May 2019) to obtain clean reads for the subsequent de novo assembly through Trinity Software (https://github.com/trinityrnaseq/trinityrnaseq/wiki, accessed on 30 May 2019). After processing with the TransRate software (http://hibberdlab.com/transrate/, accessed on 30 May 2019), any redundant sequences were removed, and unique transcript fragments (unigenes) were generated. A BLAST search of the unigenes against several databases was conducted, including the non-redundant protein database (NR), the Swiss-Prot database, the protein families database (Pfam), the Clusters of Orthologous Groups database (COG), the Gene Ontology database (GO), and the Kyoto Encyclopedia of Genes and Genomes database (KEGG).

### 2.9. Analysis of Differentially Expressed Genes

The software RSEM v1.3.3 was used to record the mapping results and to quantify gene expression. The results of gene abundance were normalized and correlated with the TPM (Transcript per million reads) value. The DESeq2 1.26.0 software was used for screening differentially expressed genes (DEGs), with fold change (FC) ≥ 2, FC ≤ 2, and *p* value < 0.05 as screening criteria. These DEGs were displayed with volcanic maps. Then all downregulated DEGs with |Log_2_FC| ≥ 2 were screened and their distribution in KEGG pathways was categorized to explore the function of the most significantly downregulated DEGs. Finally, representative KEGG pathways and relative DEGs were further explored.

### 2.10. Validation of RNA Sequencing Data Using Quantitative RT-PCR (qPCR)

To further validate the gene expression results from the RNA sequencing data, fifteen differently expressed unigenes were randomly selected for q-PCR analysis. The genes included Claudin 10, Myosin 11, Myosin 10, Caspase 3, Cathepsin L, Cathepsin H, alpha-Tubulin 8, etc. Firstly, we conducted reverse transcription of all RNA samples by using the Eastep^®^ RT Master Mix Kit (Promega, Beijing, China), and then the cDNA were collected for the follow-up qPCR experiment. The Primer premier 5 software (Premier Biosoft International, San Francisco, CA, USA) was applied to design the gene-specific qPCR primers, as shown in Table S1. An amount of 18S mRNA was selected as a housekeeping gene [26]. The qPCR experiment was performed in a 10 μL reaction volume, containing 0.2 μL of forward primer, 0.2 μL of reverse primer, 1 μL of cDNA template, 3 μL of nuclease-free water, and 5 μL of 5 × SYBR^®^ Premix Ex Taq, according to the manual of the ChamQ Universal SYBR qPCR Master Mix kit (Vazyme, Nanjing, China), on an Eppendorf Mastercycler^®^ ep Realplex4 instrument (Hamburg, Germany), with the following program: denaturation at 95 °C for 2 min, followed with 40 cycles of 95 °C for 10 s, and annealing at 60 °C for 10 s and at 72 °C for 15 s. The relative gene expression was calculated and normalized with the 2^−ΔΔCt^ method [28]. All PCR reactions were repeated three times.

### 2.11. Statistical Analysis

The growth indices, serum biochemical parameters, and gene expression were analyzed with the One-way analyses of variance (ANOVA) and the Tukey’s test using SPSS 23.0 (Chigaco, IL, USA). The results are presented as mean ± SE, with *p* < 0.05 indicating a significant difference.

## 3. Results

### 3.1. Growth Performance, Antioxidant Capacity, and Non-Specific Immunity Indices

After 8 weeks of PC supplementation, the PC group showed no significant differences in growth when compared with the control group (*p* > 0.05). The SSI and HSI of groupers from different groups showed no significant differences either (*p* > 0.05) (Table S2).

The antioxidant T-AOC and CAT indices of the PC group were both significantly lower than those in the control group. There was no significant difference in the amounts of SOD. Significantly higher amounts of ALP and LZM were observed in the PC group (Table 1).

### 3.2. De Novo Assembly and Sequence Annotation

All raw reads were uploaded and deposited in the National Center for Biotechnology Information (NCBI, PRJNA9008813). After filtration, 73.93 Gb of clean reads, with error rate < 0.025%, Q20 > 98%, Q30 > 94%, and about 48% GC of content (Table S3), were assembled to obtain a total of 67,825 unigenes from 10 samples (Table 2). The length distribution of the unigenes showed that almost 60% of the unigenes were clustered in the range of 0–1500 bp (Figure S1).

### 3.3. Functional Annotation

A total of 67,825 unigenes were annotated in six databases (Nr, Swiss-Prot, Pfam, COG, GO, KEGG), among which 26,864 unigenes were annotated in at least one of the six databases. There were 26,128 (38.52%), 20,629 (30.42%), 18,636 (27.48%), 11,718 (17.28%), 7745 (11.42%), and 16,156 (23.82%) unigenes annotated in the Nr, Swiss-Prot, Pfam, COG, GO, and KEGG databases, respectively (Table 2).

The Nr annotation revealed that the top five species showing the highest similarity with the intestine transcriptome of *E.fuscoguttatus* were *Lates calcarifer* (4893, 18.97%), *Larimichthy crocea* (4622, 17.92%), *Seriola dumerili* (2727, 10.57%), *Seriola lalandi* (1837, 7.12%), and *Stegastes partitus* (1269, 4.92%) (Figure S2).

The GO annotation revealed that a total of 34,561 unigenes were annotated in three GO categories: molecular function, cellular component, and biological process (Figure S3). In the molecular function category, binding (3790, 42.37%) and catalytic activity (3223, 36.03%) were the two classes with the most annotations; in the cellular component category, most unigenes were annotated in the classes of cell (2364, 18.57%), cell part (2324, 18.25%), membrane (2281, 17.91%), and membrane part (2082, 16.35%); and in the biological process category, most unigenes were annotated in the classes of cellular process (2820, 21.74%), metabolic process (1976, 15.23%), and single-organism process (1925, 14.84%).

In addition, the annotation in the KEGG pathways showed that the majority of annotated unigenes belonged to six of KEGG’s first categories (Signal transduction, Cancers: Overview, Immune system, Endocrine system, Transport and catabolism, and Infectious diseases: Viral), which contained twenty of KEGG’s second categories (Figure S4).

### 3.4. Identification and Annotation of DEGs in the E. fuscoguttatus Intestinal Transcriptome in Response to PC Supplementation

**Figure 1:**
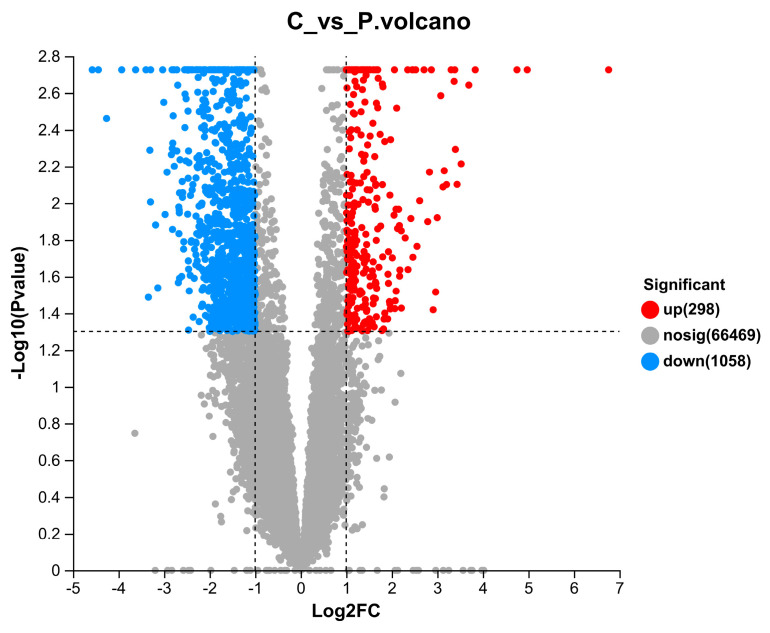
Volcano plot of DEGs.

**Figure 2:**
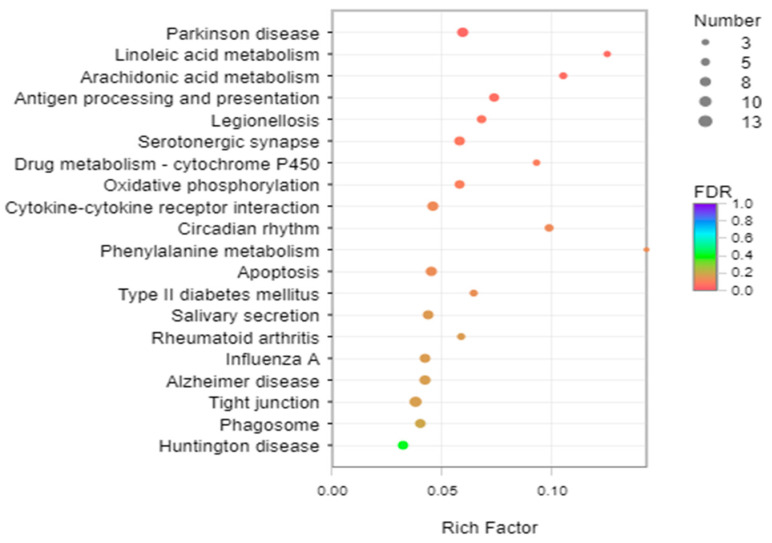
KEGG pathway enrichment analysis of the annotated DEGs. The *Y*-axis represents the KEGG pathway; the *X*-axis represents the rich factor. The dot size represents the number of DEGs of the pathway, and the dot color represents the q-values.

When the samples of the PC group were compared to those of the control group, a total of 1356 DEGs, including 298 upregulated DEGs (with an elevated expression in the PC group) and 1058 downregulated DEGs (with a decreased expression in the PC group) were identified (as shown in Figure 1). The top 20 most significant enriched KEGG pathways (sub-categories, as shown in Figure 2) belonged to five KEGG categories: human diseases (7 pathways); metabolism (5 pathways); organismal systems (4 pathways); cellular processes (3 pathways), and environmental information processing (1 pathway). In particular, the highest numbers of DEGs belonged to pathways related to epithelium integrity (Tight junction, 13 DEGs), apoptosis (Apoptosis, 11 DEGs; Parkinson’s disease, 11 DEGs; Alzheimer’s disease, 11 DEGs; and Huntington’s disease, 10 DEGs), and immune regulation (Cytokine–cytokine receptor interaction, 11 DEGs; Antigen processing and presentation, 9 DEGs; Phagosome, 10 DEGs; Influenza A, 10 DEGs; Legionellosis, 8 DEGs; and Rheumatoid arthritis, 6 DEGs). Representative enriched DEGs included caspase-3, cathepsin B, cathepsin L, claudin-11, claudin-1, and 14-3-3 protein beta/alpha-1 (Table 3).

### 3.5. Validation of 15 Putative DEGs using qPCR

**Figure 3:**
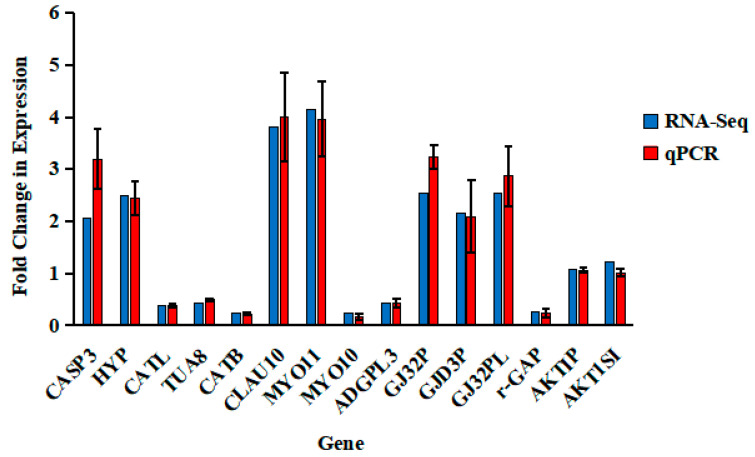
Validation of the RNA-Seq results and comparison of the gene expression between the control group and the PC supplementation group of *E. fuscoguttatus* using qPCR. The expressions of genes were examined relative to 18S RNA expressions as the endogenous control gene. The expression values were normalized against those of the control group. The results are the mean ± SEM of intestine tissues derived from five individual fish (*n* = 3). CASP3: Caspase 3; HYP: Hypothetical protein EH28_09075; CATL: Cathepsin L; TUA8: Tubulin alpha-8; CTSB: Cathepsin B; CLAU-10: Claudin 10; MYO11: Myosin 11; MYO10: Myosin 10; ADGPL3: Adhesion G protein-coupled receptor L3; GJ32P: gap junction Cx32.2 protein; GJD3P: gap junction delta-3 protein; GJ32PL: gap junction Cx32.2 protein-like; rGAP: ras GTPase-activating-like protein; AKTIP: AKT-interacting protein; and AKT1S1: proline-rich AKT1 substrate 1.

The gene expression of 15 randomly selected DEGs, including Claudin 10, Myosin 11, Myosin 10, Caspase 3, Cathepsin L, Cathepsin H, Tubulin-alpha 8, etc, were analyzed. The comparative results are shown in Figure 3. It is evident that the RNA sequencing data is valid and the transcriptome analysis is credible.

### 3.6. Disease Resistance

**Figure 4:**
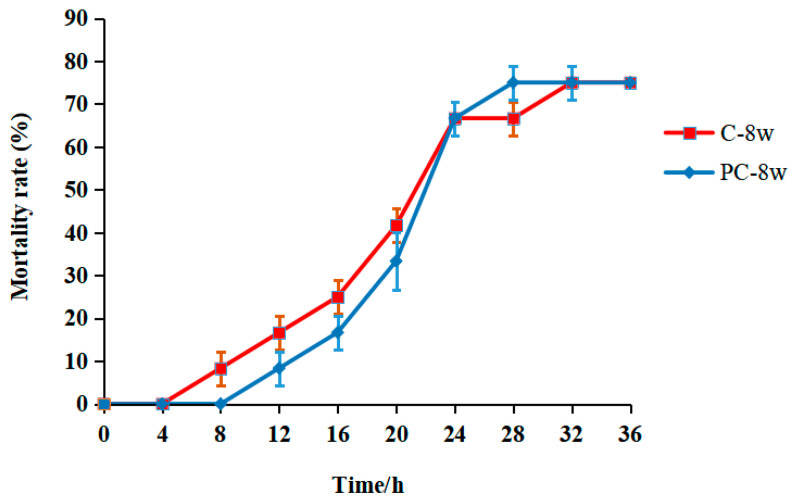
Cumulative mortality (%) of *E. fuscoguttatus* fed with PC for 8 weeks after the Vibrio harveyi challenge. Abbreviations: C, control group; and PC, Phellodendron chinese cortex fed group. Note: Data are expressed as the mean ± SD (*n* = 3). No significant difference between groups (*p* > 0.05).

Due to the presence of strain GDH11385 [27], though mortality was observed for 7 days after the challenge, Figure 4 only shows cumulative mortality records from 0 to 36 h, because fish deaths only occurred within the first 36 h.

The 8-week mortality rate was recorded, and the comparison charts are displayed in Figure 4. The challenge results showed that 8 weeks of supplementation with the PC water extract caused no significant differences in disease resistance against *Vibrio harveyi* in tiger groupers.

## 4. Discussion

As a traditional Chinese medicine, Phellodendri chinese cortex is commonly used in combination with other traditional Chinese herbs to treat inflammatory disease, diarrhea, dysentery, and fatty liver disease, and to help with weight reduction [29,30]. Studies of phellodendri Chinese cortex used as an immunopotentiator by itself are rare. To our best knowledge, the only study of the phellodendri Chinese cortex in fish was Cai et al. [26], in which the short-term (14-day) supplementation with PC was found to be immunosuppressive and to potentially enhance the disease resistance of *E. fuscoguttatus* against *Vibrio harveyi* significantly [26].

Catalase (CAT) and the total antioxidant capacity (T-AOC) are both important antioxidant parameters. Widely distributed in tissues, CAT can exercise an antioxidant function through degradation of hydrogen peroxide [31]. The T-AOC aggregates all antioxidants and is applied mostly for scavenging ROS and iNOS [32]. In this present study, the CAT and the T-AOC of the grouper serum significantly declined after 8 weeks of supplementation with PC. This indicated that antioxidant enzymes and antioxidant non-enzymatic molecules significantly reduced in response to oxidative stress. These changes were different from the results of Cai et al. [26]. Chen et al. [33] found that the T-AOC in muscle was significantly reduced in Rhynchocypris that were fed highly oxidized fish oil compared with a fresh fish oil group after 4 weeks. Lysome (LZM) and Alkaline phosphate (ALP) are widely perceived as immune markers in serum. Lysozyme (LZM) is a crucial enzyme that disturbs the pathogens’ cytoderm, whereas ALP is a classical hydrolase. The increased amount of LZM and ALP in serum normally suggests enhanced immunity in aquatic organisms [34]. The amount of LZM and ALP in the serum of groupers increased significantly after 8 weeks of supplementation with PC. This is different from Cai et al. [26], who found no significant differences in ALP activity after 1 week or 2 weeks of PC supplementation to the diet of groupers. In summary, the long-term exposure to PC seemed to decrease the antioxidant capacity of tiger groupers and to enhance the non-specific immunity of tiger groupers.

The intestinal tract is not only involved in food digestion and absorption but it also plays an important role in immune, metabolic, and osmotic regulation [35,36]. The intestine transcriptome DEG analysis of the control vs. the PC group revealed that the top 20 pathways in the KEGG database mainly involved three major categories: neurodegeneration and apoptosis; immune and infectious diseases; and endocrine, digestion, and metabolism.

Apoptosis is a form of programmed cell death that is critical to physiologic homeostasis in almost every organ system. The Caspase family plays an important role in apoptosis activation and executions, especially in the mRNA and protein expression of caspase 3, which is an essential regulator of programmed cell death. The Caspase 3 gene encodes a protein that triggers cell death through cleaving specific proteins in the cytoplasm and nucleus. Cell apoptosis is induced by various biotic factors, such as bile acid disorder [37], and various abiotic factors, such as Carnitine [38], alcohol, radiotherapy, severe burns [39], etc. Previous studies of Caspase 3 in aquatic organisms revealed that in grass carp (*Ctenopharyngodon idella*) [40] and turbot (*Scophthalmus maximus* (L.) [41] long-term stress and pathogen infection could both bring about organ damage by inducing an apoptosis response through a significant increase in the expression of caspase 3 in the spleen. In our research, the qPCR and RNA-Seq results both indicated that the mRNA expression of caspase 3 was significantly increased after 8 weeks of PC supplementation. This result is also in agreement with Eissa et al. [42], who studied berberine, the major component of PC, in rats. Eissa et al. [42] reported that the Caspae-3 mRNA level was significantly upregulated in rats that received berberine (50 mg/kg/day) via oral gavage for 6 consecutive weeks [42]. Contrary to the pro-apoptosis effects of the long-term administration of PC and berberine, several studies [43,44] have shown that the short-term administration of berberine has significant anti-apoptosis effects. For example, a 7-day treatment of berberine (50 mg/kg or 200 mg/kg) induced significantly downregulated caspase-3 in mice (*p* < 0.01) [43]; a 10-day administration of berberine (5 mg/mL) resulted in a significantly decreased expression of the pro-apoptosis caspase-3 genes in the epithelial cells of mice [44]. These results indicate that the long-term administration of PC might induce damage to the physical barrier of healthy tiger groupers’ intestines through activation of the apoptosis process.

Generally speaking, when the apoptosis response increases, it may be accompanied by changes in other cellular processes of organisms [45]. The DEG analysis of this present study showed that there were a pronounced number of DEGs, which indicated impaired epithelium integrity and increased permeability in the intestines of tiger groupers. For example, our RNA-seq data demonstrated that the expression of Claudin 10 was upregulated and the expression of Claudin 11 was downregulated. These two genes both belong to the claudin family (which are integral membrane proteins and components of tight junction strands) yet have opposite functions. While the expression of Claudin-10 promotes the tight junction cation pore in the intestinal epithelium (to increase permeability), the expression of Claudin 11 enhances barrier integrity, because the Claudin 11 protein is an obligatory protein for tight junction formation [46,47]. In the intestinal tract, the overexpression of Claudin 10 or the insufficient expression of Claudin 11 is usually induced by pathogen infection and considered as a sign of intestinal damage [48]. This damaging effect of long-term PC supplementation on intestine cell structure and permeability was also reflected by the downregulation of many other DEGs that are relevant to structural constituents, such as tubulin alpha-1B (TUBA1B) chain-like, tubulin alpha-8 chain-like, myosin light polypeptide 6, 14-3-3 protein beta/alpha-1, and integrin alpha-6-like. Alpha tubulins are a core protein family that form microtubules. TUBA1B is a structural constituent of the cytoskeleton, which is important in maintaining a stable backbone in both axons and dendrites. Lower levels of TUBA1B were observed in patients with schizophrenia (a serious mental disorder), compared to controls [49]. TUBA1B is also positively correlated with the process of DNA replication and mitotic cell cycle. In mice, the TUBA8 mRNA/protein was significantly higher in the fatty liver and tumor tissues of mice and in hepatocarcinoma (liver cancer) patients [50,51]. The downregulation of the TUBA1B gene in normal groupers reflects both the damaging effect on intestinal integrity and the potential anti-fatty liver and anti-cancer effects of long-term PC supplementation. Myosin light polypeptide 6 is a structural constituent of muscle, regulating the function of myosin. Downregulated myosin gene expressions induce decreased levels of myosin, which may compromise the mitochondrial structure and consequently its function, leading to the loss of cellular energetics, an increase in the amount of free radicals, and eventually cell death [52]. 14-3-3 proteins were expected to play a significant role in cellular osmoregulation. Expression of the 14-3-3 beta/alpha-1 transcripts in fish were most abundant in the gill and to a lesser extent in the intestine. The downregulation of this gene in PC-supplemented groupers indicated the impaired function of osmotic regulation [53].

The anti-inflammatory effect of PC as a traditional herbal medicine used to treat various infectious or inflammatory diseases has long been recognized. In this present study, both the transcriptome and RT-PCR analysis revealed that the PC exhibited strong downregulating effects on the immune- and inflammation-associated genes and pathways of tiger groupers’ intestines. In particular, along with many other immune-associated genes (HSPA1_8 and HSPA90A, for example), the cathepsin B and cathepsin L genes (CTSBs and CTSLs) were significantly downregulated in the PC-supplemented group. CTSBs and CTSLs, with similar structures and functions, are ubiquitously expressed lysosomal cysteine proteinases that play important roles in inflammation, infection, and cancer, where they are highly expressed [54]. In channel catfish infected with *Edwardsiella Ictaluri* and grouper fish challenged with Singapore grouper iridovirus and *Vibrio anguillarum*, strongly upregulated CTSBs and CTSLs were observed along with many other pro-inflammatory genes in the intestines [55,56]. Studies have shown that the activation of CTSB and CTSL gene expression after infection is associated with the destruction of elastin-rich tissues during inflammatory responses, and that the inhibition of CTSBs and CTSLs could effectively ameliorate the mucosal damage of inflammatory diseases such as colitis [57]. Therefore, in addition to its anti-inflammatory effects, the supplementation with PC also exhibited a potential cathepsin inhibition effect, which might be helpful in the prevention and therapy of fish enteritis [57].

Like many medicinal herbs, PC has an optimum dietary intervention period to exhibit the best anti-*Vibrio* effect in grouper fish. Cai et al. [26] and this present study show that, while 7-day and 56-day supplementation with PC did not significantly improve the disease resistance of tiger groupers against *V. harveyi*, the 14-day supplementation with PC significantly decreased the cumulative mortality of tiger groupers after the *V. harveyi* challenge. Therefore, the disease resistance-improving effect of PC varied with different supplementation periods. The trial design had some limitations. In future, we need to consider the PC dose treatment, the sampling time, and the challenge dose to verify the effect of the PC.

## 5. Conclusions

This study demonstrated that a long-term (8-weeks) PC diet did not affect the growth performance of tiger groupers, significantly increased the ALP and LZM activity in grouper serum, and had no significant effect on the disease resistance of tiger groupers. The transcriptome analysis revealed that long-term PC supplementation to groupers’ diet may potentially damage the structural and functional integrity of healthy groupers’ intestines. On the other hand, long-term PC supplementation also exhibited potential anti-inflammatory and cathepsin inhibition effects, which might be helpful for the prevention and therapy of fish enteritis.

## Figures and Tables

**Table 1:** Statistics of the antioxidant capacity and immunity in serum.

	T-AOC	CAT	SOD	ALP	LZM
C	7.04 ± 1.42 *	47.42 ± 17.16 *	19.75 ± 4.03	41.19 ± 30.05	112.65 ± 21.52
PC	4.59 ± 1.28	33.763 ± 28.02	21.45 ± 3.59	107.59 ± 24.68 **	222.65 ± 24.50 **

The results are presented as mean ± SE (*n* = 5), and different asterisks above each figure mean significant differences in the Tukey’s test (*p* < 0.05). Control: control group; and PC: Phellodendri Cortex group. * *p* < 0.05; and ** *p* < 0.01.

**Table 2:** Statistics of the transcriptomic sequences from six libraries.

Database	Unigene Number	Percentage
NR	26,128	38.52%
Swiss-Prot	20,629	30.42%
Pfam	18,636	27.48%
COG	11,718	17.28%
GO	7745	11.42%
KEGG	16,156	23.82%
Total annotated unigenes	26,864	39.61%
Total unigenes	67,825	100.00%

**Table 3:** Selected DEGs involved in the PC stimulation of *E. fuscoguttatus*.

Gene Id	Gene Description	Gene Symbol	KEGG Categories	Log2(FC)	Regulate
**Apoptosis/Immune responses**					
TRINITY_DN21229_c6_g3	cathepsin B-like	CTSB	Immune system; Cell growth and death	−2.2	down
TRINITY_DN15782_c3_g2	cathepsin L, partial	CTSL1	Immune system; Cell growth and death	−1.3	down
TRINITY_DN16713_c4_g5	cathepsin L, partial	CTSL2	Immune system; Cell growth and death	−1.2	down
TRINITY_DN18426_c2_g3	heat shock protein 90 beta	HSP90B	Immune system; Cardiovascular disease; Endocrine system	−2.6	down
TRINITY_DN20083_c0_g1	caspase-3-like	CASP3	Cell growth and death; Immune system; Infectious disease: viral; Infectious disease: bacteria	1.0	up
**Epithelium integrity**					
TRINITY_DN24936_c0_g1	claudin-11	CLAU-11	Cellular community-eukaryotes; Immune system; Signaling molecules and interaction; Infectious disease: viral	−2.8	down
TRINITY_DN20497_c0_g2	14-3-3 protein beta/alpha-1	14-3-3PB/A1	Aging; Signal transduction; Cell growth and death; Cancer: overview; Infectious disease: viral	−2.2	down
TRINITY_DN13254_c0_g2	myosin light polypeptide 6	MYOLP6	Cellular community-eukaryotes; endocrine system; circulatory system	−1.7	down
TRINITY_DN19287_c0_g2	tubulin alpha-1B chain-like	TUA-1B	Cell growth and death; Cellular community- eukaryotes; Infectious disease: bacterial; Transport and catabolism	−1.6	down
*TRINITY_DN19417_c2_g1	integrin alpha-6-like	IL-6	Signal transduction; Signaling molecules and interaction; Cellular community-eukaryotes; Immune system; Cancer: overview	−1.3	down
TRINITY_DN19287_c0_g3	tubulin alpha-8 chain-like	TUA8	Cell growth and death; Cellular community- eukaryotes; Infectious disease: bacterial; Transport and catabolism	−1.2	down
TRINITY_DN9279_c0_g2	claudin-10-like isoform X1	CLAU-10	Cellular community-eukaryotes; Immune system; Signaling molecules and interaction; Infectious disease: viral	1.9	up

## Data Availability

All data generated or analyzed during this study are included in this article. Raw data are available from the authors upon reasonable request. RNA-seq data have been deposited in the Sequence Read Archive (SRA) APP. The accession code is PRJNA908813.

## Supplementary Materials

The following supporting information can be downloaded at: https://www.mdpi.com/article/10.3390/life13122336/s1, Table S1. Primers used for qPCR; Table S2. Effects of PC diet on growth performance of tiger grouper after 8-week feeding trial; Table S3. Statistics of unigenes assembled; Table S4. Statistics of annotation results for intestine transcriptome unigenes; Figure S1. The length distribution of the unigenes; Figure S2. The species distribution based on Nr annotation; Figure S3. Top 47 annotated GO terms in the intestine transcriptome of *E. fuscoguttatus*; Figure S4. Annotated KEGG pathways in the intestine transcriptome of *E. fuscoguttatus*.

## References

[1] Sudirman, Halide H., Jompa J., Zulfikar, Iswahyudin, McKinnon A.D. (2009). Wild fish associated with tropical sea cage aquaculture in South Sulawesi, Indonesia. Aquaculture.

[2] De Benedetto G., Arfuso F., Ferrara M.C., Brianti E., Gaglio G. (2021). Parasite Fauna of the Dusky Grouper (*Epinephelus marginatus*, Lowe 1834) from the Central Mediterranean Sea. Animals.

[3] Hoihuan A., Soonson P., Bunlipatanon P., Thawonsuwan J., Tanasomwang V., Areechon N., Unajak S. (2021). Molecular genotyping and phenotyping of *Vibrio vulnificus* isolated from diseased, brown-marbled grouper (*Epinephelus fuscoguttatus*) in Thailand with vaccine. Aquaculture.

[4] Noor N.M., Defoirdt T., Alipiah N., Karim M., Daud H., Natrah I. (2019). Quorum sensing is required for full virulence of *Vibrio campbellii* towards tiger grouper (*Epinephelus fuscoguttatus*) larvae. J. Fish Dis..

[5] Yang Y., Lin H., Xing Y., Wen J., Fang W., Zhang J., Duan Y. (2022). Response signatures of intestinal microbiota and gene transcription of the tiger grouper *Epinephelus fuscoguttatus* to nervous necrosis virus infection. Aquaculture.

[6] Wang C., Yao Z., Zhan P., Yi X., Chen J., Xiong J. (2023). Significant tipping points of sediment microeukaryotes forewarn increasing antibiotic pollution. J. Environ. Sci..

[7] Xia Y.T., Wu Q.Y., Cheng E.H.C., Dong T.T.X., Qin Q.W., Wang W.X., Tsim K.W.K. (2022). The inclusion of extract from aerial part of Scutellaria baicalensis in feeding of pearl gentian grouper (*Epinephelus fuscoguttatus* × *Epinephelus lanceolatus*) promotes growth and immunity. Fish Shellfish. Immunol..

[8] Zhou X., Wang Y., Yu J., Li J., Wu Q., Bao S., Jiang L., Liu B. (2022). Effects of dietary fermented Chinese herbal medicines on growth performance, digestive enzyme activity, liver antioxidant capacity, and intestinal inflammatory gene expression of juvenile largemouth bass (*Micropterus salmoides*). Aquac. Rep..

[9] Cheng M.E., Zhan Z.L., Zhang W., Yang H.J., Shen J.Z., Peng H.S. (2019). Textual research of “Huangbo” in classical prescriptions. Zhongguo Zhong Yao Za Zhi.

[10] Ding X., Tang Y., Sun A., Liu R. (2015). Simultaneous determination of three alkaloids in Huangbo using an ionic liquid as a mobile phase additive in reversed-phase liquid chromatography. J. Sep. Sci..

[11] Liu C.S., Zheng Y.R., Zhang Y.F., Long X.Y. (2016). Research progress on berberine with a special focus on its oral bioavailability. Fitoterapia.

[12] Takei Y. (2021). The digestive tract as an essential organ for water acquisition in marine teleosts: Lessons from euryhaline eels. Zool. Lett..

[13] Chang X., Liu P., Feng J., Su X., Huang M., Chen Y., Zhang J., Li B. (2020). Impact of chronic exposure to the ionic liquid ([C8mim] [PF6]) on intestinal physical barrier, immunological barrier and gut microbiota in common carp (*Cyprinus carpio* L.). Environ. Res..

[14] Li H., Zhou Y., Ling H., Luo L., Qi D., Feng L. (2019). The effect of dietary supplementation with Clostridium butyricum on the growth performance, immunity, intestinal microbiota and disease resistance of tilapia (*Oreochromis niloticus*). PLoS ONE.

[15] Gerile S., Pirhonen J. (2017). Replacement of fishmeal with corn gluten meal in feeds for juvenile rainbow trout (*Oncorhynchus mykiss*) does not affect oxygen consumption during forced swimming. Aquaculture.

[16] Amoah K., Yan X., Liu H., Pan S., Li T., Suo X., Tan B., Zhang S., Huang W., Xie M. (2022). Substituting fish meal with castor meal in diets of hybrid grouper (*Epinephelus fuscoguttatus* ♀ × *E. lanceolatus* ♂): Effects on growth performance, immune response, antioxidant and digestive enzyme activities, gut morphology, and inflammatory-related gene expression. Fish Shellfish. Immunol..

[17] Yong A.S.K., Syed Mubarak N.S., Zhuo L.C., Lin Y.H., Shapawi R. (2022). Oxidized Palm Oil Diet Affects Fatty Acid Profiles, Apparent Digestibility Coefficients and Liver of Hybrid Grouper Juvenile (*Epinephelus fuscoguttatus* × *Epinephelus lanceolatus*). Front. Sustain. Food Syst..

[18] Ismail R., Yong A.S.K., Lim L.S., Kawamura G., Shapawi R. (2018). Utilization of different dietary carbohydrate sources in hybrid grouper, Tiger grouper (*Epinephelus fuscoguttatus*, ♀) × Giant grouper (*Epinephelus lanceolatus*, ♂) juveniles. Int. J. Aquat. Sci..

[19] Zuo Z., Wang S., Wang Q., Wang D., Wu Q., Xie S., Zou J. (2022). Effects of partial replacement of dietary flour meal with seaweed polysaccharides on the resistance to ammonia stress in the intestine of hybrid snakehead (*Channa maculatus* ♀ × *Channa argus* ♂). Fish Shellfish. Immunol..

[20] Wang S., Li X., Zhang M., Jiang H., Wang R., Qian Y., Li M. (2021). Ammonia stress disrupts intestinal microbial community and amino acid metabolism of juvenile yellow catfish (*Pelteobagrus fulvidraco*). Ecotoxicol. Environ. Saf..

[21] Gomez D., Sunyer J.O., Salinas I. (2013). The mucosal immune system of fish: The evolution of tolerating commensals while fighting pathogens. Fish Shellfish. Immunol..

[22] Nardocci G., Navarro C., Cortés P.P., Imarai M., Montoya M., Valenzuela B., Jara P., Acuña-Castillo C., Fernández R. (2014). Neuroendocrine mechanisms for immune system regulation during stress in fish. Fish Shellfish. Immunol..

[23] Rosengren M., Thörnqvist P.-O., Winberg S., Sundell K. (2018). The brain-gut axis of fish: Rainbow trout with low and high cortisol response show innate differences in intestinal integrity and brain gene expression. Gen. Comp. Endocrinol..

[24] Groschwitz K.R., Hogan S.P. (2009). Intestinal barrier function: Molecular regulation and disease pathogenesis. J. Allergy Clin. Immunol..

[25] Martín R., Laval L., Chain F., Miquel S., Natividad J., Cherbuy C., Sokol H., Verdu E.F., Vlieg J.v.H., Bermudez-Humaran L.G. (2016). *Bifidobacterium animalis* ssp. lactis CNCM-I2494 Restores Gut Barrier Permeability in Chronically Low-Grade Inflamed Mice. Front. Microbiol..

[26] Cai Y., Wang S., Guo W., Xie Z., Zheng Y., Cao Z., Zhou Y. (2018). Transcriptome analysis provides insights into the immune responsive pathways and genes in the head kidney of tiger grouper (*Epinephelus fuscoguttatus*) fed with Spatholobus suberectus, Phellodendron amurense, or Eclipta prostrata. Fish Shellfish. Immunol..

[27] Xu X., Liu K., Wang S., Guo W., Xie Z., Zhou Y. (2017). Identification of pathogenicity, investigation of virulent gene distribution and development of a virulent strain-specific detection PCR method for *Vibrio harveyi* isolated from Hainan Province and Guangdong Province, China. Aquaculture.

[28] Livak K.J., Schmittgen T.D. (2001). Analysis of relative gene expression data using real-time quantitative PCR and the 2^−ΔΔCT^. Methods.

[29] Lee E., Yoon S.H., Kim H., Kim Y.D., Leem J., Park J. (2020). Ephedrae Herba in combination with herbal medicine (Zhizichi decoction and Phellodendri Cortex) for weight reduction: A case series. Integr. Med. Res..

[30] Xu B., Yan Y., Huang J., Yin B., Pan Y., Ma L. (2020). Cortex Phellodendri extract’s anti-diarrhea effect in mice related to its modification of gut microbiota. Biomed. Pharmacother..

[31] Sohal R.S., Ku H.-H., Agarwal S., Forster M.J., Lal H. (1994). Oxidative damage, mitochondrial oxidant generation and antioxidant defenses during aging and in response to food restriction in the mouse. Mech. Ageing Dev..

[32] Qiao Y., Sun J., Ding Y., Le G., Shi Y. (2013). Alterations of the gut microbiota in high-fat diet mice is strongly linked to oxidative stress. Appl. Microbiol. Biotechnol..

[33] Chen X., Wang Q., Guo Z., Zhao Y., Gao Y., Yu T., Chen Y., Zhang D., Wang G. (2019). Effects of dietary oxidized fish oil on growth performance and antioxidant defense mechanism of juvenile *Rhynchocypris lagowski* Dybowski. Aquaculture.

[34] Yin G., Li W., Lin Q., Lin X., Lin J., Zhu Q., Jiang H., Huang Z. (2014). Dietary administration of laminarin improves the growth performance and immune responses in *Epinephelus coioides*. Fish Shellfish. Immunol..

[35] Morrison D.J., Preston T. (2016). Formation of short chain fatty acids by the gut microbiota and their impact on human metabolism. Gut Microbes.

[36] Peterson L.W., Artis D. (2014). Intestinal epithelial cells: Regulators of barrier function and immune homeostasis. Nat. Rev. Immunol..

[37] Lack L., Suliman H.B., Rahman A.A., Abou-Donia M.B. (2005). Cholestyramine feeding lowers number of colonic apoptotic cells in rat. J. Toxicol. Environ. Health A.

[38] Ye J., Han Y., Chen X., Xie J., Liu X., Qiao S., Wang C. (2014). L-Carnitine attenuates H_2_O_2_-induced neuron apoptosis via inhibition of endoplasmic reticulum stress. Neurochem. Int..

[39] Wang K. (2014). Molecular mechanisms of hepatic apoptosis. Cell Death Dis..

[40] Yuan Z., Feng L., Jiang W., Wu P., Liu Y., Kuang S., Tang L., Zhou X. (2021). Dietary choline deficiency aggravated the intestinal apoptosis in association with the MAPK signalling pathways of juvenile grass carp (*Ctenopharyngodon idella*). Aquaculture.

[41] Pan M., Liu D., Liu J., Li X., Huang D., Luo K., Liu Y., Wu Z., Zhang W., Mai K. (2022). Biotin alleviates hepatic and intestinal inflammation and apoptosis induced by high dietary carbohydrate in juvenile turbot (*Scophthalmus maximus* L.). Fish Shellfish. Immunol..

[42] Eissa L.A., Kenawy H.I., El-Karef A., Elsherbiny N.M., El-Mihi K.A. (2018). Antioxidant and anti-inflammatory activities of berberine attenuate hepatic fibrosis induced by thioacetamide injection in rats. Chem.-Biol. Interact..

[43] Shen Y., Liu Y.C., Wang Z.L., Ruan X.L., Li S., Ni S.Y., Zhong J.H. (2020). Effect of berberine from Coptis chinensis on Apoptosis of Intestinal Epithelial Cells in a Mouse Model of Ulcerative Colitis: Role of Endoplasmic Reticulum Stress. Evid.-Based Complement. Altern. Med..

[44] Fang C.Z., Xie L.L., Liu C.M., Fu C.H., Ye W., Liu H., Zhang B.H. (2018). Berberine ameliorates neonatal necrotizing enterocolitis by activating the phosphoinositide 3-kinase/protein kinase B signaling pathway. Exp. Ther. Med..

[45] Levine A.D., Fiocchi C. (2001). Regulation of life and death in lamina propria T cells. Semin. Immunol..

[46] Reiner J., Berlin P., Wobar J., Schäffler H., Bannert K., Bastian M., Vollmar B., Jaster R., Lamprecht G., Witte M. (2020). Teduglutide Promotes Epithelial Tight Junction Pore Function in Murine Short Bowel Syndrome to Alleviate Intestinal Insufficiency. Dig. Dis. Sci..

[47] Wu P., Tian L.I., Zhou X.Q., Jiang W.D., Liu Y., Jiang J., Xie F., Kuang S.Y., Tang L., Tang W.N. (2018). Sodium butyrate enhanced physical barrier function referring to Nrf2, JNK and MLCK signaling pathways in the intestine of young grass carp (*Ctenopharyngodon idella*). Fish Shellfish. Immunol..

[48] Ji X., Qiao Y., Zheng W., Jiang H., Yao W. (2021). Deoxynivalenol interferes with intestinal motility via injuring the contractility of enteric smooth muscle cells: A novel hazard to the gastrointestinal tract by environmental toxins. Ecotoxicol. Environ. Saf..

[49] Snelleksz M., Dean B. (2021). Lower levels of tubulin alpha 1b in the frontal pole in schizophrenia supports a role for changed cytoskeletal dynamics in the aetiology of the disorder. Psychiatry Res..

[50] Hu X., Zhu H., Chen B., He X., Shen Y., Zhang X., Chen W., Liu X., Xu Y., Xu X. (2022). Tubulin Alpha 1b Is Associated with the Immune Cell Infiltration and the Response of HCC Patients to Immunotherapy. Diagnostics.

[51] Rein-Fischboeck L., Pohl R., Haberl E.M., Zimny S., Neumann M., Eisinger K., Weiss T.S., Krautbauer S., Buechler C. (2017). Tubulin alpha 8 is expressed in hepatic stellate cells and is induced in transformed hepatocytes. Mol. Cell. Biochem..

[52] Sultana R., Baglioni M., Cecchetti R., Cai J., Klein J.B., Bastiani P., Ruggiero C., Mecocci P., Butterfield D.A. (2013). Lymphocyte mitochondria: Toward identification of peripheral biomarkers in the progression of Alzheimer disease. Free. Radic. Biol. Med..

[53] Liu Z., Zhang J., Ma A., Wang X., Sun Z., Cui W., Yuan C., Zhu C. (2020). Molecular characterization, expression analysis of 14-3-3 beta/alpha and the effect of RNA interference on ion transporter protein Na^+^-K^+^-ATPase, Na^+^-H^+^-exchanger and CFTR in turbot (*Scophthalmus maximus*). Comp. Biochem. Physiol. Part B Biochem. Mol. Biol..

[54] Gobec S., Frlan R. (2006). Inhibitors of cathepsin B. Curr. Med. Chem..

[55] Liang J.Z., Rao Y.Z., Lun Z.R., Yang T.B. (2012). Cathepsin L in the orange-spotted grouper, *Epinephelus coioides*: Molecular cloning and gene expression after a *Vibrio anguillarum* challenge. Fish Physiol. Biochem..

[56] Wei S., Huang Y., Huang X., Cai J., Yan Y., Guo C., Qin Q. (2014). Characterization of cathepsin B gene from orange-spotted grouper, *Epinephelus coioides* involved in SGIV infection. Fish Shellfish. Immunol..

[57] Menzel K., Hausmann M., Obermeier F., Schreiter K., Dunger N., Bataille F., Falk W., Scholmerich J., Herfarth H., Rogler G. (2006). Cathepsins B, L and D in inflammatory bowel disease macrophages and potential therapeutic effects of cathepsin inhibition in vivo. Clin. Exp. Immunol..

