# Supplementary material for: Long-Term Phellodendri Cortex Supplementation in the Tiger Grouper (Epinephelus fuscoguttatus): Dual Effects on Intestinal Health Revealed by Transcriptome Analysis
Source: Life (Basel). 2023 Dec 13;13(12):2336. doi: 10.3390/life13122336 (PMC10745030; doi:10.3390/life13122336)
Supplement: Supplementary file 1 [file life-13-02336-s001.zip › life-2742836-supplementary.pdf]

Supplementary materials

Table S1. Primers used for qPCR.

Gene ID	Forward primer(5′→3′)	Reverse primer(5′→3′)
AKTIP	GGCAAAGGAGCGGATGTTC	CGGCTTCACCCAAGACAGA
AKT1S1	TGTTGCCAAGTTGCTGGTGAT	GTGGGAGAATGTAGAATACGTCTGA
CASP3	CTGGGCTTCAAGGTGGACAT	CCCGTGAGACGACAAGACAG
HYP	GGGAGGAAGATGACAGGATGG	CCCGAAGTTTACGACCGATTT
CATB	ACGTCCGACTTCATGCACTACAC	ACGTCCGACTTCATGCACTACAC
CATL	GCCATCTTATCTGTCTGCTCAA	ACAGTTGCTTTGTAACGGATTCTC
TUA8	CGATCACAAGTTCGACCTCATG	ACCCAAATCCTCGTAATCCCT
CLAU-10	TCATCACCACAGCGACCTT	CCAGCATCGAGGGAAAGTC
MYO11	TATTCGCCAGGCCAACACT	GCCATCAGGAAACGATAGGAG
MYO10	TGTTCAGGGTGGGTCAGAGTAA	GGAAGCGTATAATGGTGTCAGTG
ADGPL3	ACTTCATTTGGAGTTTCATAGGACC	ACCCACGATTTGATGTTGTCC
GJ32P	TCTTTATGTTGGCAATGTCCG	CTCTGAACTGCTGTTCTTCCTTTT
GJD3P	GGTTACCGCCCAGGACTACA	GAAAGCCAATCTCCGCCAC
GJ32PL	CCTCTTTGGCACATACATAACC	GGCACAGGGTGGAGACATT
rGAP	GGTGTTTGACATCGTTCCTGC	CTTCTTCGTACCTGGTATTTGAGG
18 S	CGGTGGTACTTTCTGTGC	TGTGGTAGCCGTTTCTCA

Table S2. Effects of PC diet on growth performance of tiger grouper after 8-week feeding trial.

Item	C	PC
SSI, %	0.13 ± 0.05^{a}	0.12 ± 0.04^{a}
HSI, %	0.188 ± 0.41^{a}	1.62 ± 0.5^{a}
SGR, %*day^{-1}	3.67 ± 0.75^{a}	3.45 ± 0.17^{a}
WGR, %	35.12 ± 1.92^{a}	33.08 ± 2.12^{a}

SSI: Spleen somatic indices; HSI: hepatopancreas somatic indices; SGR: Specific growth rate; WGR: Weight gain rate; Data was presented as mean ± SE. Values with different superscripts in the same row were significantly different ($P < 0.05$). The lack of superscript letter indicated no significant differences among groups ($P > 0.05$).

Table S3. Statistics of unigenes assembled.

Type	Resource
Total unigenes number	67825
Largest unigene length(bp)	22824
Smallest unigene length(bp)	201
Average unigene length(bp)	1369.6
[a]N50	2173
[b]E90N50	2750
GC percent	45.17
Mean mapped reads	1883.606843

[a] : The assembled unigenes were sorted according to the length, N50 represents the length of the corresponding transcript when the length of unigenes is accumulated to half of the total length; [b] : The unigenes with the top 90% expression were sorted according to the length. E90N50 represents the length of the corresponding transcript when the length of the transcript is accumulated to half of the total length.

Table S4. Statistics of annotation results for intestine transcriptome unigenes.

Sample	Clean reads	Clean bases	Error rate (%)	Q20(%)	Q30(%)	GC content (%)
C_1	42868780	6402362666	0.0243	98.33	94.82	48.2
C_2	50759038	7554392639	0.0241	98.4	95	47.46
C_3	45485416	6789238074	0.0246	98.21	94.48	47.98
C_4	49994434	7458281090	0.0242	98.37	94.9	48.71
C_5	46643140	6953372256	0.0241	98.4	95	48.27
PC_1	56396278	8419828751	0.0241	98.41	95.02	48.69
PC_2	49936814	7450822733	0.0241	98.4	95.01	49.36
PC_3	48444140	7220277276	0.0239	98.48	95.2	48.56
PC_4	55973024	8354387035	0.0242	98.36	94.87	48.11
PC_5	49105230	7326443055	0.0244	98.29	94.71	48.53

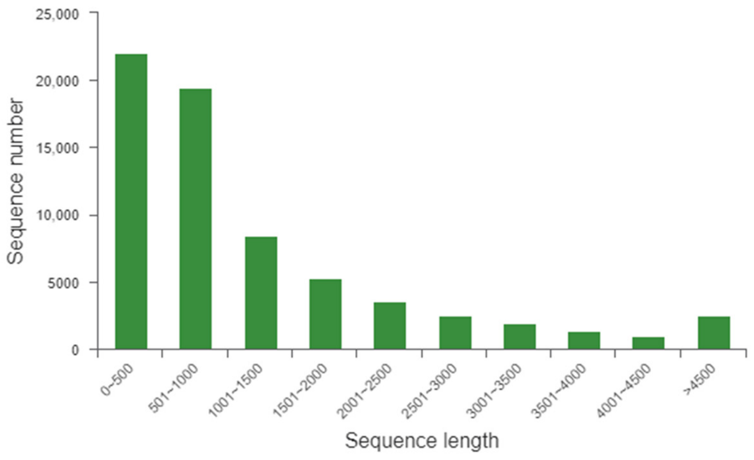

Figure S1. The length distribution of the unigenes.

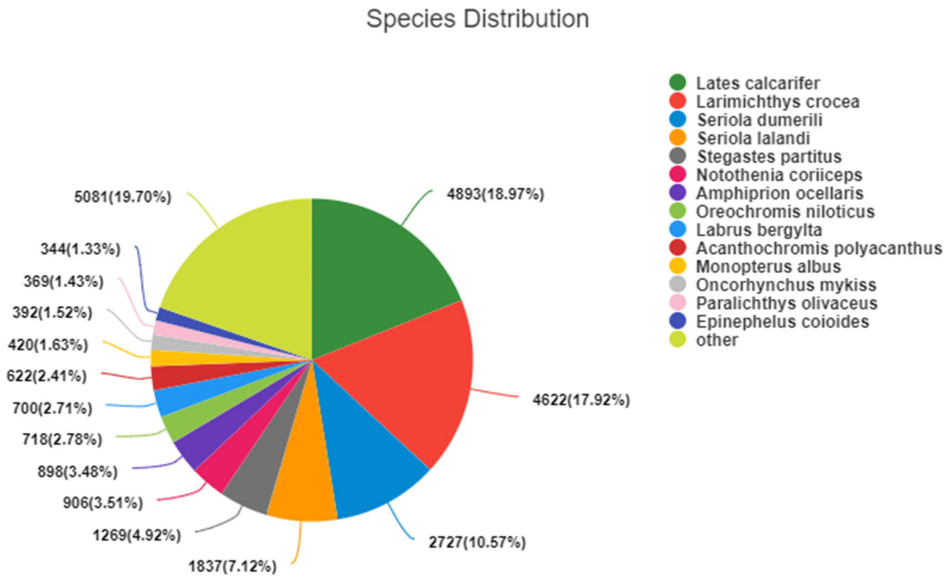

Figure S2. The species distribution based on Nr annotation.

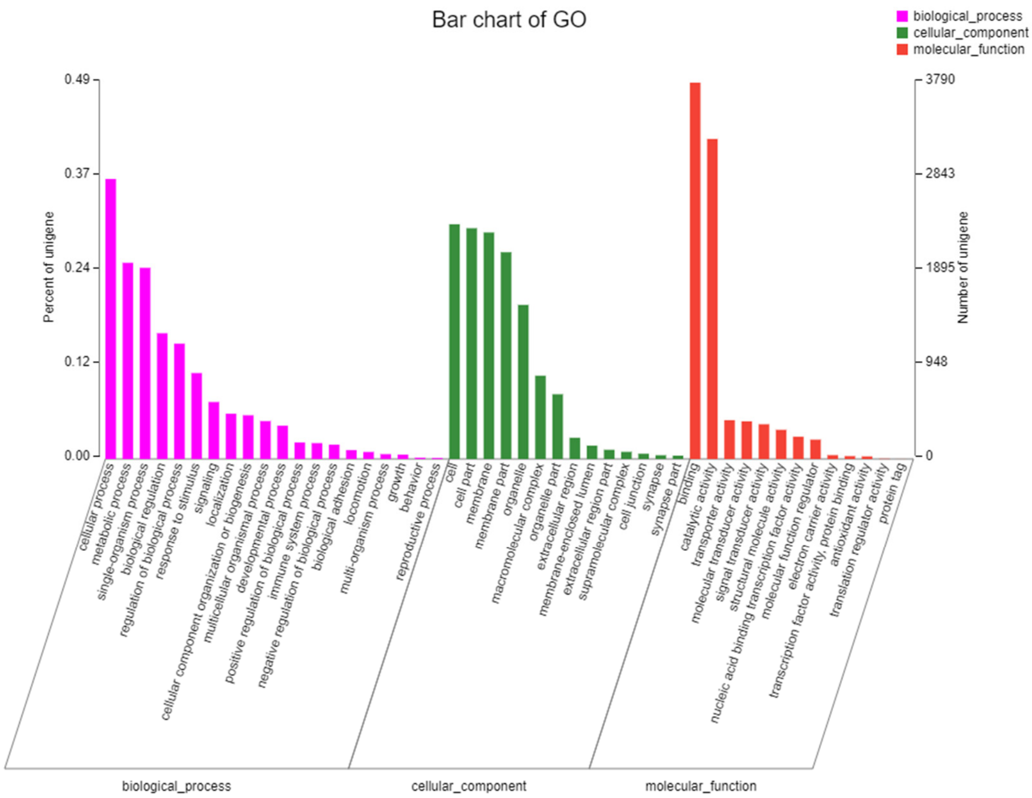

Figure S3. Top 47 annotated GO terms in the intestine transcriptome of *E. fuscoguttatus.*

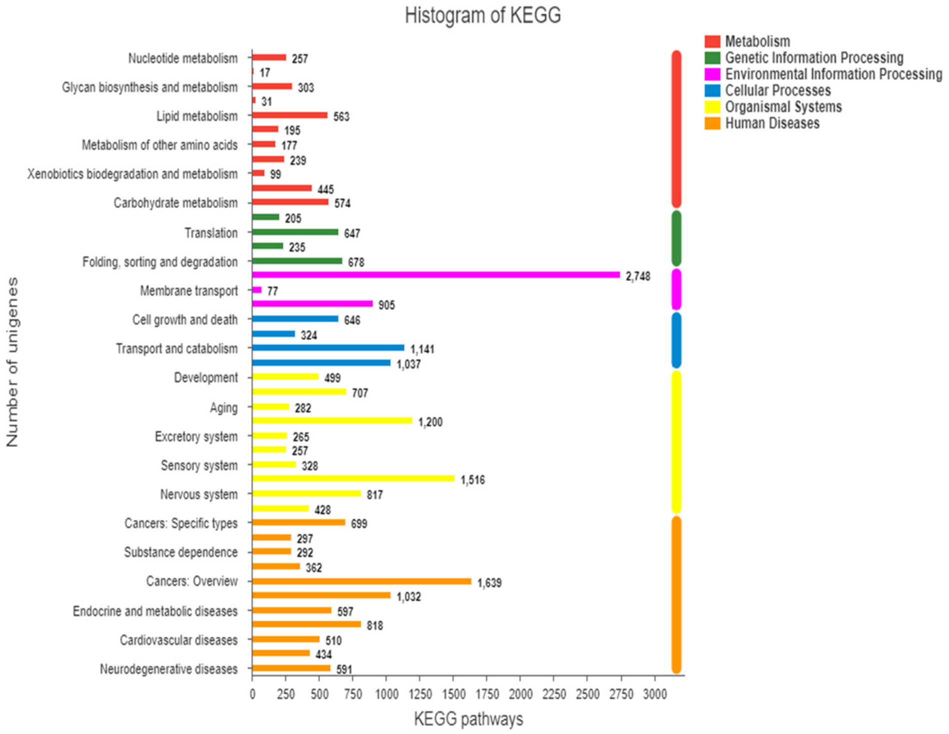

Figure S4. Annotated KEGG pathways in the intestine transcriptome of *E. fuscoguttatus.*
